# Single Gene Mutations in *Pkd1* or *Tsc2* Alter Extracellular Vesicle Production and Trafficking

**DOI:** 10.3390/biology11050709

**Published:** 2022-05-06

**Authors:** Prashant Kumar, Fahad Zadjali, Ying Yao, Michael Köttgen, Alexis Hofherr, Kenneth W. Gross, Darshan Mehta, John J. Bissler

**Affiliations:** 1 Department of Pediatrics, Le Bonheur Children’s Hospital, University of Tennessee Health Science Center, Memphis, TN 38103, USA; prashant.kumar@fda.hhs.gov (P.K.); fahadz@squ.edu.om (F.Z.); yyao@uthsc.edu (Y.Y.); 2 Children’s Foundation Research Institute (CFRI), Le Bonheur Children’s Hospital, Memphis, TN 38103, USA; 3 US FDA National Center for Toxicological Research, Jefferson, AR 72079, USA; darshan.mehta@fda.hhs.gov; 4 Department of Clinical Biochemistry, College of Medicine & Health Sciences, Sultan Qaboos University, Muscat 123, Oman; 5 Renal Division, Department of Medicine, Medical Center, Faculty of Medicine, University of Freiburg, 79106 Freiburg, Germany; michael.koettgen@uniklinik-freiburg.de (M.K.); alexis.hofherr@uniklinik-freiburg.de (A.H.); 6 CIBSS—Centre for Integrative Biological Signaling Studies, 79104 Freiburg, Germany; 7 Department of Molecular and Cellular Biology, Roswell Park Comprehensive Cancer Center, Buffalo, NY 14263, USA; kenneth.gross@roswellpark.org; 8 Pediatric Medicine Department, St. Jude Children’s Research Hospital, Memphis, TN 38105, USA

**Keywords:** extracellular vesicles (EVs), autosomal dominant polycystic kidney disease (ADPKD), tuberous sclerosis complex (TSC), primary cilia, cystogenesis, renal cyst

## Abstract

**Simple Summary:**

Extracellular vesicles shed from primary cilia may be involved in renal cystogenesis. The disruption of the *Pkd1* gene in our cell culture system increased the production of EVs in a similar way that occurs when the *Tsc2* gene is disrupted. Disruption of the primary cilia depresses EV production, and this may be the reason that the combined *Kif3A*/*Pkd1* mutant mouse has a less severe phenotype than the *Pkd1* mutant alone. We initiated studies aimed at understanding the renal trafficking of renally-derived EVs and found that single gene disruptions can alter the EV kinetics based on dye tracking studies. These results raise the possibility that EV features, such as cargo, dose, tissue half-life, and targeting, may be involved in the disease process, and these features may also be fertile targets for diagnostic, prognostic, and therapeutic investigation.

**Abstract:**

Patients with autosomal dominant polycystic kidney disease (ADPKD) and tuberous sclerosis complex (TSC) are born with normal or near-normal kidneys that later develop cysts and prematurely lose function. Both renal cystic diseases appear to be mediated, at least in part, by disease-promoting extracellular vesicles (EVs) that induce genetically intact cells to participate in the renal disease process. We used centrifugation and size exclusion chromatography to isolate the EVs for study. We characterized the EVs using tunable resistive pulse sensing, dynamic light scattering, transmission electron microscopy, and Western blot analysis. We performed EV trafficking studies using a dye approach in both tissue culture and in vivo studies. We have previously reported that loss of the *Tsc2* gene significantly increased EV production and here demonstrate that the loss of the *Pkd1* gene also significantly increases EV production. Using a cell culture system, we also show that loss of either the *Tsc2* or *Pkd1* gene results in EVs that exhibit an enhanced uptake by renal epithelial cells and a prolonged half-life. Loss of the primary cilia significantly reduces EV production in renal collecting duct cells. Cells that have a disrupted *Pkd1* gene produce EVs that have altered kinetics and a prolonged half-life, possibly impacting the duration of the EV cargo effect on the recipient cell. These results demonstrate the interplay between primary cilia and EVs and support a role for EVs in polycystic kidney disease pathogenesis.

## 1. Introduction

During the last decade, inherited renal cystic disease has been the fourth leading cause of end-stage renal disease in the United States [1]. Among the best-recognized inherited polycystic kidney diseases are autosomal dominant polycystic kidney disease (ADPKD), occurring in about 1:1000 people [2], and autosomal recessive polycystic kidney disease (ARPKD), occurring in 1:20,000 [3]. A less recognized, though common, cystic kidney disease that is also inherited in an autosomal dominant fashion is tuberous sclerosis complex (TSC), occurring in 1:5800 [4]. Approximately 70% of the TSC patient population exhibit one of five different patterns of renal cystic disease [5], one of which is a severe polycystic kidney disease variety [4]. The autosomal dominantly inherited polycystic kidney diseases, ADPKD and TSC, are distinct from ARPKD because patients with the former diseases have normal or near-normal kidneys at birth and acquire the cystic phenotype with time, while ARPKD is a developmental disorder occurring in utero. Understanding the biology of these dominantly inherited diseases could lead to treatments that sustain the near-normal kidney phenotype by initiating therapy early in the course of disease.

These two autosomal dominantly inherited polycystic kidney diseases, ADPKD and TSC, have an interesting relationship genetically. About 70% of TSC cases are linked to the *TSC2* gene located on chromosome 16p13.3, and about 85% of ADPKD cases are linked to the *PKD1* gene, which is also located on 16p13.3 immediately adjacent to the *TSC2* gene. The remaining TSC cases are associated with the *TSC1* gene located on chromosome 9q34, and the remaining ADPKD cases are linked to the *PKD2* gene located on chromosome 4q21. The disease severity is greater, and the onset is earlier for *PKD1-* and *TSC2-*linked diseases than for the diseases linked to the *TSC1* gene or the *PKD2* gene. The *TSC2* gene location was originally identified through studies involving a family with ADPKD caused by a balanced translocation involving the *PKD1* gene. The identified proband had both typical ADPKD and TSC due to an unbalanced translocation [6]. In contrast, the contiguous deletion of both the *TSC2* and *PKD1* genes has a severe and very early onset polycystic kidney disease phenotype [7]. Why patients with contiguous gene deletions have far more severe disease than that associated with independent *TSC2* and *PKD1* mutations remains an enigma [8]. 

The disease mechanism responsible for the development of cystic disease in ADPKD and TSC is unclear. A characteristic of ADPKD is that, although all cells in a patient carry a heterozygous germline mutation, only about 0.05% of nephrons are involved in the disease process [9]. This focal nature of the disease was attributed to a two-hit model before the causative genes were even identified [10]. Following the identification of the *PKD* genes, work supported a possible role for somatic mutation for both loci [11,12,13,14]. Though this second hit mechanism has been suggested [15], this mechanism for ADPKD is not straightforward because cysts can still express the polycystin protein, suggesting that a threshold for polycystin expression may also be involved in cyst development [16]. Specifically, the complete loss of the *Pkd* gene function may not be required, and levels below a threshold of expression can lead to cystogenesis [17,18,19]. The loss of the *Pkd1* gene in mouse cysts likewise seems to confer a license for cystogenesis in adjacent nephrons through an unknown signaling system [20]. Other factors, including developmental timing, participate in the cellular choreography of cystogenesis [21]. Although TSC-associated angiomyolipomata exhibit a ‘second hit’ at the involved *TSC* locus [22] with loss of protein expression [23], the mechanism for TSC cystic disease is nuanced, given that the majority of renal cysts in a mouse model maintain their *Tsc* locus integrity [24,25] and human TSC cysts continue to express both tuberin and hamartin [26]. 

There is a strong association of renal cystic disease with primary cilia. In the renal collecting duct, primary cilia are microtubular-based organelles that extend from the apical surface of the principal cell as hair-like structures. They are expressed on almost every cell and function during development to affect nodal flow to facilitate the embryonic left–right axis determination. Primary cilia have a life-long function in sensing extracellular and intracellular signals that influence cell proliferation, differentiation, and tissue maintenance [27,28]. There are numerous renal ciliopathy syndromes, including ARPKD, ADPKD, nephronophthisis, and Bardet–Biedl syndrome, all of which phenotypically exhibit renal cystic disease [29]. Although TSC is not a ciliopathy, the TSC proteins, like many of the proteins linked to ciliopathy diseases, have a role in tubule morphogenesis through planar cell polarity [30] and cilia formation [31,32]. The *TSC* gene products regulate the mechanistic target of rapamycin complex 1 (mTORC1) activity [33]. The cystic epithelium in both ADPKD [34,35,36] and TSC [4] exhibit increased mTORC1 activity, and such increased activity is reported to impact polycystin-1 trafficking [8,37,38]. 

Primary cilia also interact with extracellular vesicles (EVs) such that the cilia function as both EV transmitters and receivers [39,40,41,42,43,44,45]. Extracellular vesicles are lipid-bound structures of varying sizes that carry cargo consisting of RNA, protein, lipid, and even DNA and are produced both through the endosomal pathway and by budding from the plasma membrane [46]. Ciliary EVs have significant implications for renal cystic disease. Primary cilia are expressed on renal tubular principal cells, but rarely on intercalated cells [47]. Targeted disruption of the *Pkd1* gene in principal cells leads to significant renal cystic disease, while such disruption in intercalated cells leads to a muted phenotype [48]. Further demonstrating the complex interplay between the primary cilia and cystic disease, either the disruption of *Pkd1* or loss of primary cilia result in similar cystic phenotypes, while simultaneous inactivation of the *Pkd1* gene and primary cilia result in a *decreased cystic phenotype* compared with the *Pkd1* knockout [49]. 

We have recently demonstrated that in vivo disruption of the *Tsc1* [50] or *Tsc2* [51] gene in renal principal cells conscripted intercalated cells into cystogenesis. The loss of the *Tsc2* gene in principal cells results in significantly increased EV production, and these EVs have an altered proteosome that induces significantly different responses in target cells compared to *Tsc2* intact principal cells in a cell culture model [52]. Knocking out *Tsc2* increases the production of EVs in vivo and in vitro and augments the uptake of EVs from the *Tscs2* mutant principal cells compared to EVs from Tsc1 mutant or unmodified principal cells [53]. These findings in TSC renal cystic disease, combined with the role of ciliary EVs, support our hypothesis that ciliary EVs have an important role in renal tissue homeostasis such that proper dose, cargo, and affinity maintain the tissue, while perturbations in these EV features lead to structural diseases such as polycystic kidney disease [54]. 

Here, we report three new features of ciliary EV biology as it relates to renal cystic disease. First, we demonstrate that the disruption of the *Pkd1* gene in our cell culture system increased the production of EVs, as also occurs for *Tsc2* deletion. We reasoned that such an EV dose effect could help explain the disease severity differences between contiguous and independent deletions of *Tsc2* and *Pkd1*. The disruption of *Pkd1* in mIMCD3 cells also causes the cells to double their EV output, as occurs with the disruption of *Tsc2*. Second, we measured the effect of *Kif3A* disruption on EV production. We reasoned that if the loss of *Pkd1* increased the EV concentration and disruption of *Kif3A,* which inhibits ciliogenesis [55], lowered EV production, the resulting lowered EV dose, may explain the muted phenotype of the combined *Kif3A* and *Pkd1* deletion. Finally, we assessed the ex vivo organ trafficking activity of renally-derived EVs and assessed the effects of single-gene disruption on EV kinetics.

## 2. Results

### 2.1. Pkd1 Mutant Renal Epithelial Cells Produce More EVs Than Pkd2 Mutant Cells 

**Figure 1:**
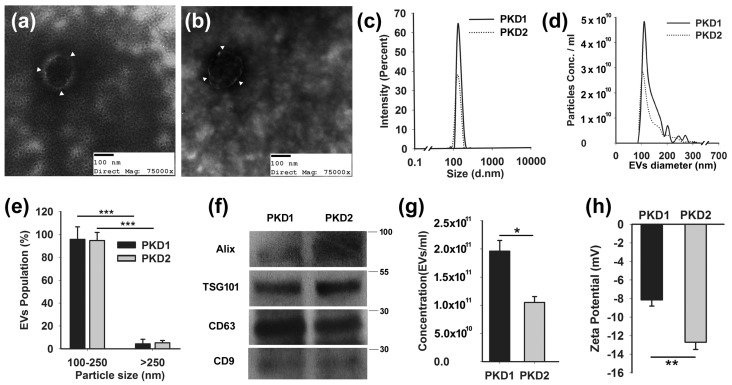
mIMCD3 cells with disrupted *Pkd1* and *Pkd2* genes produce EVs. (**a**) *Pkd1-* and (**b**) *Pkd2*-disrupted mIMCD3 cells have cup-shaped morphology when examined by transmission electron microscopy as indicated by white arrowheads. Isolated EVs were approximately 110 nm in diameter by (**c**) dynamic light scattering and (**d**) tunable resistive pulse sensing analysis. (**e**) More than 90% of EVs were less than 250 nm. (**f**) The *Pkd* mutant cell EVs expressed Alix, TSG101, CD63, and CD9, as expected for EVs. (**g**) Disruption of *Pkd1* in mIMCD3 cells caused the cells to double their EV production compared to the disruption of Pkd2 in the same cells. (**h**) The EVs from the Pkd mutant cell lines had significantly different z-potentials, indicating a charge difference in the EV surface. * *p* < 0.05, ** *p* < 0.01, *** *p* < 0.001. (Supplementary File S1 shows: Densitometry ratios of PKD1/PKD2).

There is mounting evidence that EVs are involved in renal cystic disease [53,56,57]. We posit that the EV dose, cargo, targeting mechanism, and target tissue characteristics determine the signaling impact of EVs. In order to study the role of EV dose, we used a panel of mIMCD3 cell lines that had been modified from the parental line using CRISPR/CAS9 to disrupt the *Tsc1, Tsc2, Pkd1,* or *Pkd2* genes. We have recently published that *Tsc2* disruption (*Tsc2*^Δ^) in this model system significantly increases the production of EVs compared to *Tsc1* disruption (*Tsc2*^Δ^) [53]. Although there is no perfect EV isolation technique, we found that size-exclusion column chromatography reliably yielded EV preparations with consistent characteristics that could be used in our studies [58]. To ascertain if the disruption of the *Pkd1* locus caused the cells to produce more EVs, we isolated EVs from the *Pkd* mutant cell lines. We found that *Pkd* mutant cell lines (*Pkd1*^Δ^ and (*Pkd2*^Δ^) produce EVs with a similar morphology, a representative example using transmission electron microscopy (TEM) is provided in Figure 1a,b. There was no morphological difference between the EVs produced by the *Pkd1*^Δ^ and *Pkd2*^Δ^ cell lines. In both cell lines, the isolated EVs were approximately 110 nm in diameter, as measured by both dynamic light scattering (DLS) and tunable resistive pulse sensing analysis (TRPS) (Figure 1c–e). The majority of the EVs were 100–250 nm in size, and less than 10% were larger. Although such a size is compatible with exosomes, our EV isolates could also contain small microvesicles and ectosomes. Similar to EVs from the *Tsc*-disrupted cell lines, the *Pkd* mutant cell EVs also expressed Alix, TSG101, CD63, and CD9 (Figure 1f). While ciliated renal cells express ARL13b, the EVs used in these experiments did not [59], supporting that these were true EVs. We found that the *Pkd1* locus consistently had an effect on EV production. The *Pkd1-*disrupted mIMCD3 cells produced twice as many EVs as the *Pkd2-*disrupted cells (Figure 1g). The disruption of *Pkd2* did not increase EV production. The *Pkd* locus involvement also had a significant impact on the EV surface charge, as measured by the ζ-potential. 

The EVs from the *Pkd2*-disrupted cell lines had a more negative ζ-potential than those from the *Pkd1*-disrupted cells, indicating changes on the EV surface (Figure 1h), raising the possibility that they may bind or interact differently with cells. 

### 2.2. Cilial Disruption Reduces EV Production

There is a strong, though complicated, association between renal cystic disease and primary cilia [49]. We wanted to understand the effect of primary cilia loss on EV production. We previously demonstrated that treating *Tsc2*^Δ^ cells with an mTORC1 inhibitor reduced the EV production back to the levels observed in the mIMCD3 cells with an intact *Tsc2* locus [56] and that there were a significant number of ciliary proteins in the EVs isolated from the *Tsc2*^Δ^ and parental mIMCD3 cells [52]. Given these findings, we posited that primary cilia could participate in EV production and that if this effect was robust enough, the loss of primary cilia would reduce EV production, similar to that seen with mTORC1 inhibition in the *Tsc2* system.

**Figure 2:**
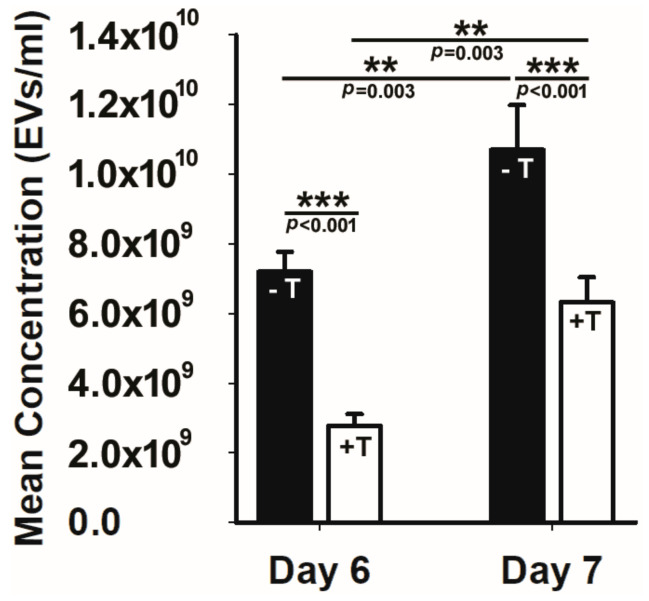
Loss of the primary cilia significantly reduced EV production. The solid black bars at days 6 and 7 post vehicle (−T) or tamoxifen treatment (+T) treatment reveal that cells treated with tamoxifen that lose their primary cilia exhibit a significant reduction in EV production. ** *p* < 0.01, *** *p* < 0.001.

To answer this fundamental question, we tested whether the loss of an anterograde intraflagellar transport protein that disrupts primary cilia would impact either EV production or cellular uptake. To achieve this, we used an immortalized renal epithelial cell line (176-5 cell line) with a conditional allele *Kif3^atm2Gsn^* that expressed a tamoxifen-inducible Cre recombinase. For these EV production and uptake experiments, we used the same cell line, adding treatment with tamoxifen to remove primary cilia (176-5Δ cell line) [60]. To obtain ciliated cells, we only added the vehicle. The cells were grown to the same, near confluence density. We found that tamoxifen treatment reduced Kif3a below detection and also reduced EV production by half compared to vehicle-treated cells (Figure 2). Because there are multiple EV production pathways, including the endosomal pathway and the plasma membrane budding pathway [46], we performed the experiment after six and seven days to see if the duration of tamoxifen treatment impacted EV production. A significant percentage reduction in EVs was present at both time points. In contrast, the loss of this anterograde intraflagellar transport protein had no effect on EV uptake when comparing EV uptake in the 176-5 and 176-5Δ cell lines. Although EVs are produced by the endosomal pathway by budding from the plasma membrane and by primary cilia, how these production pathways contribute to the EV pool is not well understood. These results demonstrate that primary cilia on renal epithelial cells are important contributors to EV production.

### 2.3. Renal EVs Home to the Kidney

We recently demonstrated that recombinase-mediated disruption of the *Tsc1* or *Tsc2* gene in principal murine cells results in cysts comprised largely of intercalated cells with intact *Tsc* genes [50,51]. We demonstrate that this cell’s nonautonomous trait is likely mediated by EVs [56]. In order for principal cell EVs to ‘conscript’ the intercalated cells into the cystic phenotype, we reasoned that cyst EVs must exhibit an addressing system possibly favoring renal homing. Studying such homing is complicated because EVs have multiple functions, including cellular clearance. The clearance of plasma EVs is rapid and mediated largely by the liver and, to a lesser degree, the spleen [61,62]. The mammalian liver exhibits an efficient blood clearance function mediated by the liver sinusoidal endothelial cells (LSECs) and Kupffer cells through phagocytosis and receptor-mediated endocytosis [63]. This clearance activity functions well for removing endogenous waste material, and the liver sinusoidal endothelial cells also play a role in blood clearance of exogenous ligands such as viruses and other nanoparticles [64]. This nanoparticle clearance function also extends to EVs [65]. The efficient hepatic EV clearance makes for a responsive intercellular signaling system that is not delayed by perseveration of messaging due to slow EV clearance. This aspect, though, complicates the interpretation of EV tracking studies as the hepatic signal may represent specific targeting, general clearance, or both.

In order to understand renal EV uptake, we compared renal uptake to the heart, lungs, and spleen in normal mice. We wished to examine the interstitial and cyst-derived EVs and compare them to the normal kidney interstitial EVs. The isolation of cystic EVs from *Pkd1* mutant mice posed a significant challenge because of the medullary cystic location and raised the high likelihood of contamination from interstitial EVs. To reduce this risk, we used the *Tsc2* mutant mouse because harvesting cystic fluid without interstitial EV contamination is far easier because the cysts are limited to the cortex of the kidney [51,56].

**Figure 3:**
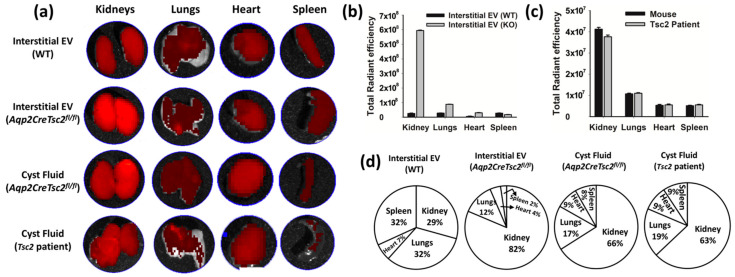
Ex vivo tissue distribution of Dir-labelled extracellular vesicles derived from mouse and human; 1.67 × 10^9^ EV per kilogram body weight were intraperitoneally injected for four hours followed by tissue excision and imaged using Perkin Elmer imaging system. (**a**) Imaging reveals the migration of EV to the kidney, lungs, heart, and spleen. (**b**,**c**) Representation of average radiant efficiency in different organs (kidney, lungs, heart, and spleen) after the introduction of EVs derived from interstitial and cyst fluid isolated from the kidney of Aqp*2CreTsc2^fl/fl^* and *Tsc2* patients. Note that due to image processing (flattening), the lung signal is actually greater than the heart. (**d**) Representation of migration of various EVs in different organs.

We used purified murine renal interstitial EVs from littermates that were identical except for the loss of *Tsc2* in the principal cell population. We also isolated the cystic EVs from the previously described *Aqp2CreTsc2^fl/fl^* cystic mouse [56] and EVs from a TSC patient with the contiguous gene syndrome. To understand EV trafficking, the EVs were stained with DIR dye, washed, and injected intraperitoneally. We compared the different EV deposition in the kidneys, lungs, heart, and spleen after 4 h (Figure 3a). These studies were performed in an ex vivo fashion, meaning the thoracic and abdominal organs were harvested, and the fluorescence intensity was measured. At this time point, the EVs were completely cleared from the plasma, as compared to untreated animal serum. We observed that renal interstitial EV from the *Aqp2CreTsc2^fl/fl^* mice homed avidly to the kidney at a significantly greater amount than the homing of interstitial EVs from mice that had intact *Tsc2* in the principal cell population (Figure 3b,d). Likewise, we observed that EVs isolated from both mouse and human cysts exhibited significant renal homing (Figure 3c,d).

### 2.4. EVs from Cultured Clonal Renal Epithelial Cell Home to Kidney

**Figure 4:**
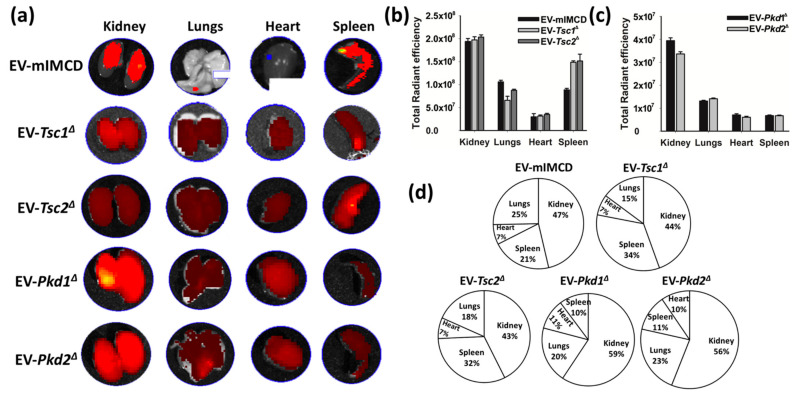
Ex vivo tissue distribution of Dir-labelled extracellular vesicles derived from cell lines; 1.67 × 10^9^ EV per kilogram body weight were intraperitoneally injected for four hours followed by tissues excision and imaged using a Perkin Elmer imaging system. (**a**) Imaging reveals the migration of EV to the kidneys, lungs, heart, and spleen. The EVs were isolated from the following cell lines: T1G (*Tsc1* deleted), T2J (*Tsc2* deleted), PKD1 (*Pkd1* deleted), and PKD2 (*Pkd2* deleted). (**b**,**c**) Representation of average radiant efficiency in different organs (kidney, lungs, heart, and spleen) after introduction of EVs purified from conditioned medium of different cell lines. (**d**) Representation of migration of various EVs in different organs. Note: Images were captured using an IVIs Perkin Elmer imaging system. The images were captured under the same instrumental settings, i.e., excitation: 750 nm, exposure time: 1000 ms, emission wavelength: 780 nm. Each image was captured under the same color scale (min = 1.00 × 10^8^; max = 1.00 × 10^9^). The data presented here is the average total radiant efficiency in the respective organs.

The impact of EVs on tissue is, at least in part, a function of their binding and entering the target cells, the amount or dose of EVs involved, their cargo, and tissue features such as the target cell transcriptome. Motivated by the finding that renally-derived EVs are home to the kidney, we use EVs derived from our panel of mIMCD3 cell lines. EVs isolated from the cell culture media demonstrated normal mouse organ uptake using the ex vivo system described earlier (Figure 4a). The tissue culture-derived EVs had a more variable hepatic clearance, making some of the renal uptakes appear different, though the fluorescent intensity of the sum of all organs was similar. Dye without EVs did not reveal a meaningful signal. The stained EVs from the parental mIMCD3 cell line, as well as from the *Tsc1*^Δ^*, Tsc2*^Δ^*, Pkd1*^Δ^*,* or *Pkd2*^Δ^ lines, revealed a significant affinity for renal tissue compared to other organs (Figure 4b,d). EVs from cells with mutations in either the *Pkd1 or Pkd2* locus also revealed significantly increased renal uptake (Figure 4c,d). The mIMCD3, *Tsc1,* and *Tsc2* were stained with a different lot of stain than that used for *Pkd1* and *Pkd2*, and thus reveals a different intensity.

### 2.5. Single Gene Defects Alter Cellular EV Uptake

**Figure 5:**
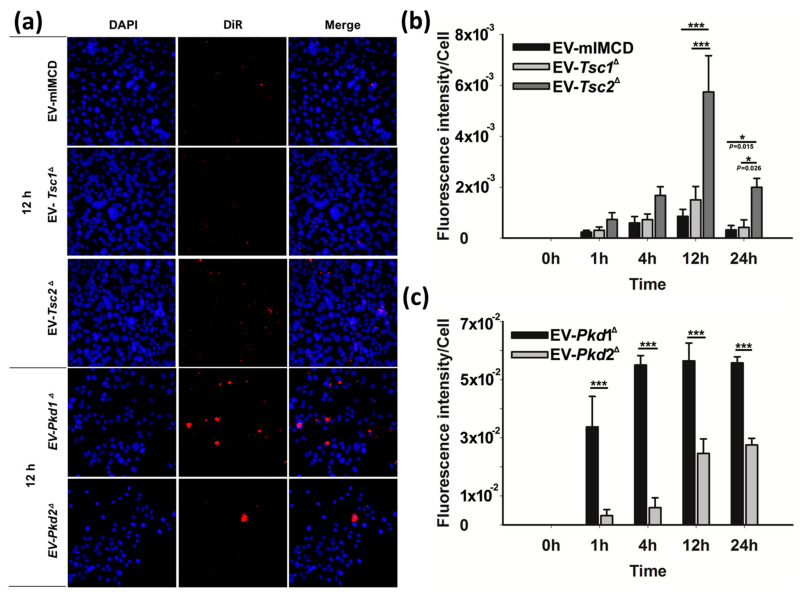
In vitro uptake of fluorescently labeled EVs derived from mIMCD, T1G, T2J, PKD1, and PKD2 cells to M1 cells. (**a**) Fluorescent EVs (5 × 10^6^) were incubated with 5 × 10^4^ M1 cells for different time points such as 0, 1, 4, 12, and 24 h. Cells were washed with cold PBS, then fixed with 4% paraformaldehyde and permeabilized with 0.1% Triton X-100. After that, cells were stained with DAPI and mounted. Images were captured with a Leica DMi8 fluorescence microscope. (**b**) Histograms represent the fluorescence intensity per cell for mIMCD, T1G, and T2J-derived EVs and (**c**) PKD1 and PKD2-derived EVs treated with M1 cells. Note—the intensity was calculated based on a minimum three images per treatment. Significance * *p* ≤ 0.05; *** *p* ≤ 0.001.

We were struck by the renal tropism of the renal epithelial cell-derived EVs. We wanted to better understand if the single gene defects altered the actual uptake kinetics of the EVs. To elucidate the effects of the single gene changes, we used the DiR fluorescent dye EVs from Figure 4 to examine the renal tubular epithelial cell uptake (Figure 5). After incubating the EVs with the M1 renal epithelial cells for the various time points, the cells were washed, the nuclei were stained with DAPI, and the cells were imaged at different time points (Figure 5a). We first examined EV internalization using the EVs from the *Tsc* mutant cell lines and found that the fluorescence per cell as a function of time was significantly different for the *Tsc2* mutant-derived EVs compared to the wild-type mIMCD and the *Tsc1* mutant-derived EVs (Figure 5b). In a separate EV staining experiment, we labeled the EVs derived from the *Pkd* mutant cell lines and again found a different time course for the fluorescence accumulation (Figure 5c). 

### 2.6. Mathematical Modeling of the EV Staining Results Offer Insight into Polycystin-1 Function

**Figure 6:**
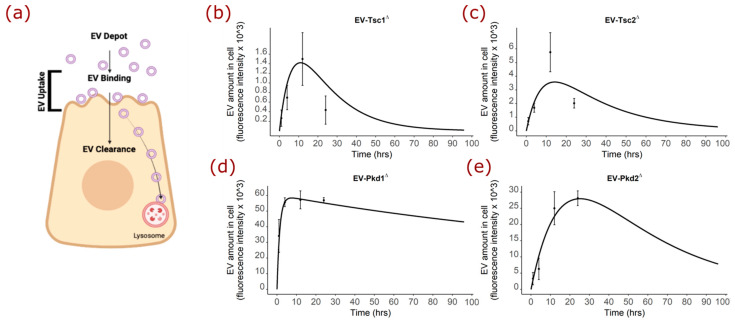
Mathematical modeling of EV-cellular interaction. (**a**) Cartoon depicting the components and phases of fluorescently labeled EV interaction with the renal cell. (**b**) Modeling of experimentally derived time course data for EVs from mIMCD3 cells with disrupted *Tsc1*, *Tsc2*, *Pkd1*, and *Pkd2* genes. (**c**) Modeling data graphed over time for EVs from mIMCD3 cells with disrupted *Tsc2*. (**d**) Modeling data graphed over time for EVs from mIMCD3 cells with disrupted *Pkd1*. (**e**) Modeling data graphed over time for EVs from mIMCD3 cells with disrupted *Pkd2*.

Because the fluorescence time course curve shape of the mutant *Pkd1-*derived EVs was so different from that of the EVs derived from the mutant *Tsc1, Tsc2, Pkd2,* or parental mIMCD cell lines, we mathematically modeled the process to understand what the *Pkd1* gene deletion could be doing. The fluorescence of DiR dye is affected when the dye is in a lipid bilayer, so we modeled the uptake, the bound and intact EVs in the cell, and clearance as first-order processes, and the binding as a saturable process. We envisioned the pool or depot of EVs that could bind, the uptake being those EVs involved in the binding process or those intact intracellular EVs, and the clearance that would require disruption of the dye/lipid interaction, such as by lysosomal disruption (Figure 6a). To describe the first-order uptake and clearance, we used the following equations respectively:

(1) $$\mathrm{Rate}\;\mathrm{of}\;\mathrm{uptake}=k_{uptake}*\;Amount_{Depot}$$

(2) $$\mathrm{Rate}\;\mathrm{of}\;\mathrm{clearance}=-k_{clearance}*\;Amount_{Cell}$$

To describe the binding, we used the equation:

(3) $$\mathrm{Rate}\;\mathrm{of}\;\mathrm{binding}=\frac{\left(B_{max}*\;Amount_{Cell}\right)}{\left(K_{M}+Amount_{Cell}\right)}$$

where *B_max_* and *K_M_* are the binding and affinity constants, respectively, governing the saturable binding process. We reasoned that the fluorescence in the cell at any point in time would be a balance of the uptake and binding versus the clearance, mathematically described in:

(4) $$\frac{d(Amount_{Cell})}{dx}=\left(k_{uptake}*\;Amount_{Depot}\right)+\frac{\left(B_{max}*\;Amount_{Cell}\right)}{\left(K_{M}+Amount_{Cell}\right)}-\left(k_{clearnace}*\;Amount_{Cell}\right)$$

The graphical data for the EVs from the identical mIMCD3 cell line, except for the genes at the Tsc or Pkd loci, has some important differences. The shape of the fluorescence as a function of time for *Tsc1*- (Figure 6b), *Tsc2*- (Figure 6c), and *Pkd2*-derived (Figure 6e) EVs were reasonably similar, while that for *Pkd1*-derived EVs (Figure 6d) was very different.

Using the equation, we compared the optimized values for *k_uptake_*, *B_max_*, *K_M_*, and *k_clearance_* modeled out to 96 h to understand the function of the polycystin-1 protein (Table 1). Simulations were conducted using the R programming language with the *mrgsolve* package.

These results reveal that while the binding (*B_max_*_)_ is reasonably similar for the EVs from the *Pkd1* and *Pkd2* mutant cell lines, the EVs from the *Pkd1* mutant cell line exhibited an uptake process that was 14 times faster and a clearance that was five times slower than those from the *Pkd2* mutant cell line. These results suggest that the *Pkd1* gene product, polycystin-1, regulates vesicular trafficking in some way. Because EVs from cells that cannot make polycystin-1 have a faster uptake and prolonged half-life, under normal circumstances, polycystin-1 would seem to reduce uptake and facilitate lysosomal trafficking, so it would be a key element regarding the ‘effective’ cellular dose of EVs. Given that the half-life of the EVs from the *Pkd1-*deleted mIMCS3 cell line is significantly extended compared to the EVs from the *Pkd2-*deleted cell line, we also modeled the behavior of the *Tsc* mutant EVs (Table 2). The EVs from the *Tsc2* mutant mIMCD cell line were taken up twice as fast, bound twice as much, and cleared three times slower than the EVs from the *Tsc1* mutant line.

## 3. Discussion

We previously demonstrated the role of EVs in mediating renal cystic disease in the tuberous sclerosis complex [52,53,56]. More recently, Ding et al. demonstrated that exogenous EVs from patients with mutations in *PKD1* or cell lines with *PKD1* mutations induced, or at least exacerbated, the PKD phenotype in three-dimensional cell culture and in *Pkd1* mutant mouse kidneys [57]. However, EVs can be different depending on what cell makes them, how they are produced, and the conditions of producing cells or tissue. Essentially, EVs are produced by either an inward-budding within the endocytic system or an outward-budding vesicle at the plasma membrane. There are three proposed mechanisms for the endosomal system to produce EVs. The first uses the endosomal sorting complex required for transport (ESCRT) machinery, consisting of four protein complexes (ESCRT-0, -I, -II, and -III) along with accessory proteins (Alix, VPS4, and VTA-1) [66]. The second pathway within the endocytic system uses ceramide synthesis to induce vesicle curvature and budding [67]. Lastly, a third mechanism uses tetraspanin to organize specific proteins, such as premelanosome protein (PMEL), to drive vesicle formation [68,69]. The PMEL protein is the target of the antibody HMB-45 used to identify TSC-associated angiomyolipomata and can stain microscopic cysts in TSC [70]. While there is ample evidence of primary cilia-related proteins being EV cargo, the exact mechanism of renal epithelial primary cilia EV production and contribution to the tissue EV pool is unknown [54,71].

Particularly relevant to renal cystic disease, primary cilia of animals such as *C. elegans*, mice, cultured human cell lines, and even flagella of protists like Chlamydomonas also produce EVs [39,40,54,72,73,74]. Based on our *Kif3A* experiments, primary cilia in these mouse cells are responsible for a measurable contribution of EVs because, despite all the mechanisms of EV production, loss of primary cilia was sufficient to suppress EV production. The number of EVs barraging a cell, their RNA, DNA, protein and lipid cargo, the rates of binding and uptake by the cell, and the duration of their effects on the cellular machinery altered by the EVs all converge to result in the EV effects on the cell/tissue. Despite being only one of many influences, the EV dose, including the concentration presented to the target cell and the duration of effect, deserves attention. While the structural loss of cilia affects the EV concentration or depot pool, so too do single gene deletions. Loss of *Pkd1,* shown here, like the previously described loss of *Tsc2* [53], greatly increases EV production. Even with a controlled input EV dose to the cell, the induced genetically-intact cells behave in a *Tsc2*-deficient fashion [56]. The effect of loss of *Tsc2* and *Pkd1* genes on the EV production may help explain why the contiguous *PKD1/TSC2* gene syndrome is a much worse disease than that seen with single, independent *PKD1* and *TSC2* mutations. We show that this EV-mediated intercellular communication system has a significant affinity for renal tissue, even when using EVs isolated from cell culture of human-derived EVs in a murine system. These results, in combination with other model system work, reveal that the cilium acts as a specialized venue for regulated EV biogenesis and signaling [71]. Dysregulation, even as seemingly small as a change in production, may result in the induction of proliferative disease in the target tissue [75]. There even appears to be a significant change in the kidney and other tissues, as uptake of EVs from the *Tsc* and *Pkd* mutant sources. 

Both *Pkd1* and *Tsc2* mutant cells produce significantly more EVs than the parental or the *Pkd2* and *Tsc1* cell lines, and their pathogenic effects on the cells may be increased by their significantly increased uptake and half-life. These EV dose effects may impact the cell transcriptome or other signaling systems [52]. While the precise function(s) of polycystin-1 still remains an enigma, our data support a possible role in EV effector signaling. While using this dye approach is extremely useful to see where the EVs traffic in the organism, there are many questions that still remain. These studies do not track the EV cargo, but rather only the lipid container. The effect and duration of effect of the EV uptake remain unknown. The observed changes are not completely negated by this, but the role of EVs in renal cystic disease deserves much more investigation. We were surprised to find that the EV binding and uptake in the cell culture system was not entirely random; rather, it seemed some cells were more receptive than others to the EVs because they would have more than one visible EV. We are unsure if this is a cell cycle-dependent feature or if there is a form of cooperativity such that once one EV is taken up, additional EVs more easily gain access. Additional research into the possible roles of EVs in disease mediation is clearly required, but hopefully, these studies will yield insight into possible diagnostic and prognostic markers, as well as additional novel therapies to reduce the cystic disease burden.

## 4. Materials and Methods

### 4.1. Cell Culture

Four different cell lines were utilized. M1 renal collecting duct cells (ATCC# CRL-2038TM, Manassas, VA) were cultured in a 1:1 ratio of Dulbecco’s Modified Essential Medium and Ham’s F-12 Medium (DMEM/F12) with 5% fetal bovine serum (FBS) and treated with 5 µM dexamethasone. Mouse-derived inner medullary collecting duct cells (mIMCD3) were cultured in DMEM/F12 containing 10% FBS. The mutants lines derived from the mIMCD3 cell line, such as T1G (*Tsc1 deleted* [53]), T2J (*Tsc2 deleted* [53]), PKD1 (*Pkd1 deleted* [76]), and PKD2 (*Pkd2 deleted* [76]) were also cultured in DMEM/F12 with 10% FBS. All other reagents used in this study were molecular grade. Cells were maintained at 37 °C in a humidified 95% air and 5% CO_2_ atmosphere.

To assess primary cilia EV production, we used an immortalized renal epithelial cell line with a conditional allele *Kif3^atm2Gsn^* (hereafter referred to as 176-5 cells) that was generated from mice carrying both the H-2Kb-tsA58 transgene (ImmortoMouse) and the ubiquitously-expressed tamoxifen-inducible CAGG-Cre/Esr1/5Amc/J, as previously described [77,78]. These cells were generously provided by Dr. Bradley Yoder (Department of Cell Biology, University of Alabama at Birmingham, Birmingham, AL, USA). By four days after treatment, functional primary cilia are no longer identifiable in the cell culture as observed by immunohistochemistry using antibodies directed at acetylated tubulin. The cells were cultured in the presence of tamoxifen to delete *Kif3a*, a kinesis motor subunit critical for cilia function and assembly (hereafter referred to as 176-5D cells).

### 4.2. Animals

All animal procedures such as animal handling, injections, experimental procedures, anesthesia, and euthanasia were performed as per the protocol approved by the Institutional Animal Care and Use Committee (IACUC) University of Tennessee, Health Science Center, Memphis, TN, USA. Animals used in this study were acquired from the Jackson laboratory. *Aqp2CreTsc2^fl/fl^* mice were produced by cross-breeding. *Aqp2CreTsc2^fl/fl^* were observed physically for the presence of cysts in the kidney. Mice were maintained up to 19–21 weeks or until they acquired kidney cyst manifestations. We used a total of thirty wild-type Balb c mice as well as three *Aqp2CreTsc2^fl/fl^* mice in these studies to obtain multiple tracking experiments per condition.

### 4.3. EV Isolation

In this study, EVs were isolated from different sources such as cell culture media, mouse renal cyst fluid, renal interstitium, and TSC patient renal cyst fluid. All sources were subjected to a series of centrifugations to get the final 500 µL ready to load in a condition Swing bucket rotor (Cat #215) in a 4 IEC Centra MP-4R Bench, Refrigerated Centrifuge used in the preparation of all EV samples (Supplemental Figure S1). All centrifugations were conducted at 4 °C unless otherwise specified. Nunc 15 mL and 50 mL conical sterile polypropylene centrifuge tubes were used for the centrifugation. EVs were purified by commercially available size-exclusion column chromatography (qEV1 Column, IzonScientific Limited, Medford, MA, USA). Source-specific approaches were performed to get the final EV-rich sample, after which they were then further loaded onto the size-exclusion column (SEC).

#### 4.3.1. From Cell Culture Media

Four cell lines, T1G (*Tsc1 deleted* [53]), T2J (*Tsc2 deleted* [53]), PKD1 (*Pkd1 deleted* [76]), and PKD2 (*Pkd2 deleted* [76]) were grown in complete media in a T-75 flask. Once cells reached the desired confluence of 75–80%, flasks were washed three times with sterile PBS to remove the traces of serum and then replenished with fresh media without serum for 16 h. Then, the culture media were harvested and centrifuged at 2000× *g* for 10 min to remove any cell debris. The supernatant was transferred on the top of an Amicon column (Millipore, Burlington, MA, USA, Cat #UFC901008) and concentrated up to 500 µL by refrigerated centrifugation as per the manufacturer’s protocol.

#### 4.3.2. From Mouse Cyst Fluid

Nineteen to twenty-one-week-old *Aqp2CreTsc2^fl/fl^* mice were examined by palpation for the presence of cysts in the kidney. Mice were sacrificed under the influence of anesthesia using Isoflurane. The kidney was surgically excised, fluid present in the cysts was aspirated using 31G insulin syringes, and debris was removed by centrifugation at 2000× *g* for 10 min at 4 °C followed by EV isolation using SEC.

#### 4.3.3. From Human Cyst Fluid

After institutional review board approval and guardian consent, the human cyst fluid was collected from the TSC patient by surgery, and clear cyst fluids were aspirated and stored on ice before processing for EV isolation. Briefly, 15 mL of cyst fluid was centrifuged at 2000× *g* for 10 min at 4 °C to remove any debris, followed by concentration using Amicon column (Millipore, Burlington, MA, USA, Cat #UFC901008) at 5000× *g* for 28 min at 4 °C. The final 500 mL concentrated sample was applied on the top of the qEV column, and EVs were eluted as per the manufacturer’s instructions.

#### 4.3.4. From Mouse Kidney Tissue

Mouse kidneys were surgically removed from the wild-type Balb c mice as well as *Aqp2CreTsc2^fl/fl^* mice under the influence of terminal anesthesia. Kidneys were immediately processed for EV isolation. Kidneys were homogenized in tissue protein extraction (T-PER) reagent (ThermoFisher, Waltham, MA, USA, Cat #78510) in a ratio of 1:20 (tissue:reagent, *w/v*) using Fisher brand Pellet Pestle Cordless Motor (Cat #12-141-361), as per the manufacturer’s instructions. The homogenized mixture was spun for 15 min at 1200× *g* to remove the cell debris. The resultant supernatant was centrifuged again at 2000× *g* for 10 min to remove any remaining debris, followed by filtration through a 0.45 μm filter; 500 µL of supernatant was utilized for the EV-isolation [58].

### 4.4. EV Isolation and Column Setup and Operation

After obtaining 500 µL of EV-rich samples, as mentioned in the previous sections, EVs were isolated using the size-exclusion column chromatography, as per the manufacturer’s instruction. The entire isolation procedure was performed at room temperature. After setting up the column, the top cap was removed, and 500 µL of EV-rich samples were applied to the top of the column by keeping the bottom cap closed. The samples were allowed to run through the column, and EV-rich fractions were collected as per the instructions and concentrated by the concentration device.

### 4.5. Physical Characterization of Extracellular Vesicles

Isolated EVs were physically characterized by using Tunable Resistance Pulse Sensing (TRPS), Dynamic Light Scattering (DLS), and Transmission Electron Microscopy (TEM). 

TRPS: EVs were characterized for size distribution by TRPS performed using a qNano system developed by iZon science. Briefly, 35 μL of the samples were allowed to pass through the nanopore NP150 when stretched at 47 mm. The system was operated at a voltage of 0.32 mV and a pressure of 15 mbar. A minimum of 500 data points were recorded for each run, and the data were processed and analyzed using Izon Control Suite v3.0 software. 

DLS: EVs were characterized for size distribution and zeta potential analysis using DLS; 50 μL of the sample EVs were homogenized with 950 μL particle-free water and analyzed using a Zetasizer Nano-ZS (Malvern Instruments, Malvern, UK). For zeta potential measurements, a 25 μL sample of EVs was mixed with 975 μL of filtered PBS and placed in the zeta cell; samples were run in auto mode. 

TEM: The samples were prepared using a negative staining technique. Briefly, a drop of EV sample was dried on a 200 mesh Formvar/Carbon-coated grid for 25 min at room temperature, followed by staining using 2% uranyl acetate. Grids were washed with particle-free PBS to remove excess stain before visualizing with a TEM microscope (JEOL 2000EXII TEM (JEOL, Peabody, MA, USA), operated at 59 kV at the Neuroimaging Center at The University of Tennessee Health Science Center. Except for *Pkd1* and *Pkd2* cell-derived EVs, all other types of EVs have been previously characterized [53]. The size and concentration of *Pkd1* and *Pkd2*-derived EVs were characterized as per the published protocol [56].

### 4.6. EV Marker Analysis by Western Blot

The EV isolated from *Pkd1* and *Pkd2* cells were directly lysed by the addition of sodium dodecyl sulfate (SDS) loading dye. The samples were resolved by SDS polyacrylamide gel electrophoresis (PAGE) followed by transfer to a polyvinylidene difluoride (PVDF) membrane. The membranes were probed for EV markers, including the transmembrane proteins CD63 and CD9 and the intracellular proteins TSG101 and ALIX. In addition, we analyzed the EV preparation for ARL13b expression. ARL13b is critical in several primary cilia-related processes, and ciliated cells stained well for this protein, including kidney principal cells and the mIMCD3 cell line [59].

### 4.7. EV Labelling

Freshly isolated EVs were labeled using a lipophilic dye as per the manufacturer’s instruction (Catalog number# D12731, lot # 2204234, ThermoFisher Scientific). Briefly, 1 μL and 5 μL of 1,1-dioctadecyl-3,3,3,3-tetramethylindotricarbocyanine iodide (DiR) were incubated with 5 × 10^10^ and 5 × 10^12^ of EV at 37 °C for 20 min. Then, EVs were washed twice, and excess dye was removed by using 100-kDa Amicon Ultra-4 (Millipore) before being further used for in vitro and in vivo experiments.

### 4.8. EV Trafficking In Vitro

To monitor the kinetics of EV internalization in vitro, EVs were labeled as per the protocol mentioned above. M1 cells (5 × 10^4^) were grown on the round glass coverslip on 12-well plates and supplemented with 500 µL DMEM media mixed with 5 × 10^6^ labeled EVs, and incubated for a range of time points between 0 and 24 h. After completion of time points, cells were washed with cold PBS and fixed with 4% paraformaldehyde, followed by permeabilization with 0.1% Triton X-100. Cells were stained with 4′,6-diamidino-2-phenylindole (DAPI) and mounted with mounting solution (ThermoFisher Scientific). Images were captured using a Leica DMi8 fluorescence microscope. The fluorescence intensity was estimated using ImageJ software. At least 100 cells for each time point were analyzed at a magnification of 40× to determine the fluorescence measure of EV uptake (Supplemental Figure S2).

### 4.9. EV Trafficking Ex Vivo

Normal Balb c mice were used in this experiment; 1.67 × 10^9^ fluorescently-labeled EVs per kg body weight were administered by intraperitoneal injection. After 4 h, animals were euthanized, and major tissues such as the brain, heart, lungs, liver, kidneys, and spleen were excised. Fluorescence intensity of the harvested organs was measured ex vivo using Caliper Life Sciences/Xenogen IVIS Lumina imaging as per the manufacturer’s instructions. The total radiant efficiency was measured and plotted. A control was used in which DiR was incubated with phosphate-buffered saline and injected intraperitoneally without EV exposure, and after four hours, the animal was sacrificed, and the organs were analyzed. Blood was collected from the experimental animals and serum was assessed for fluorescence.

### 4.10. Statistical Analysis

All experiments were performed in triplicate unless stated otherwise. Data were presented as mean ± standard deviation. Statistics were performed using Student’s *t*-test between groups. * *p* < 0.05 was considered significant.

## 5. Conclusions

Primary cilia, as well as polycystin-1, may have a role in renal tissue morphology maintenance, as disruption of either leads to renal cystic disease. A nuanced interaction is suggested because simultaneous disruption of primary cilia and polycystin-1 results in a muted cystic phenotype. The studies presented here suggest the possibility that the ‘effective dose’ of EVs may be involved in the disease process. The ‘dose’ is likely a complex result of EV production rates, tissue half-life, targeted homing, and cargo differences. Additional studies extending these findings may clarify the biology of cystogenesis, lead to diagnostic and prognostic biomarker insights, and reveal novel therapies to reduce the cystic disease burden.

## Figures and Tables

**Table 1:** Result table for mathematical modeling of Pkd mutant-derived EV data.

Parameter	*Pkd1*	*Pkd2*	Ratio
*k_uptake_*	0.70	0.05	14
*B_max_*	0.5	0.7	0.71
*K_M_*	75	45	1.67
*k_clearance_*	0.0075	0.04	0.19
half-life (h)	92.4	17.3	5.34

**Table 2:** Result table for mathematical modeling of the *Tsc* mutant-derived EV data.

Parameter	*Tsc1*	*Tsc2*	Ratio
*k_uptake_*	0.06	0.12	0.5
*B_max_*	0.12	0.25	0.48
*K_M_*	5	15	0.33
*k_clearance_*	0.15	0.05	3
half-life (h)	4.62	13.86	0.33

## Data Availability

All the data are provided in the article.

## Supplementary Materials

The following are available online at https://www.mdpi.com/article/10.3390/biology11050709/s1, Supplementary Figure S1: EV isolation flowchart; Supplementary Figure S2: Additional Cellular uptake time points. Supplementary File S1: Raw Western PKD1 and PKD2 EV Marker.
