# Supplementary material for: Single Gene Mutations in Pkd1 or Tsc2 Alter Extracellular Vesicle Production and Trafficking
Source: Biology (Basel). 2022 May 6;11(5):709. doi: 10.3390/biology11050709 (PMC9139108; doi:10.3390/biology11050709)

Supplementary Figure S1: EV isolation flow diagram.

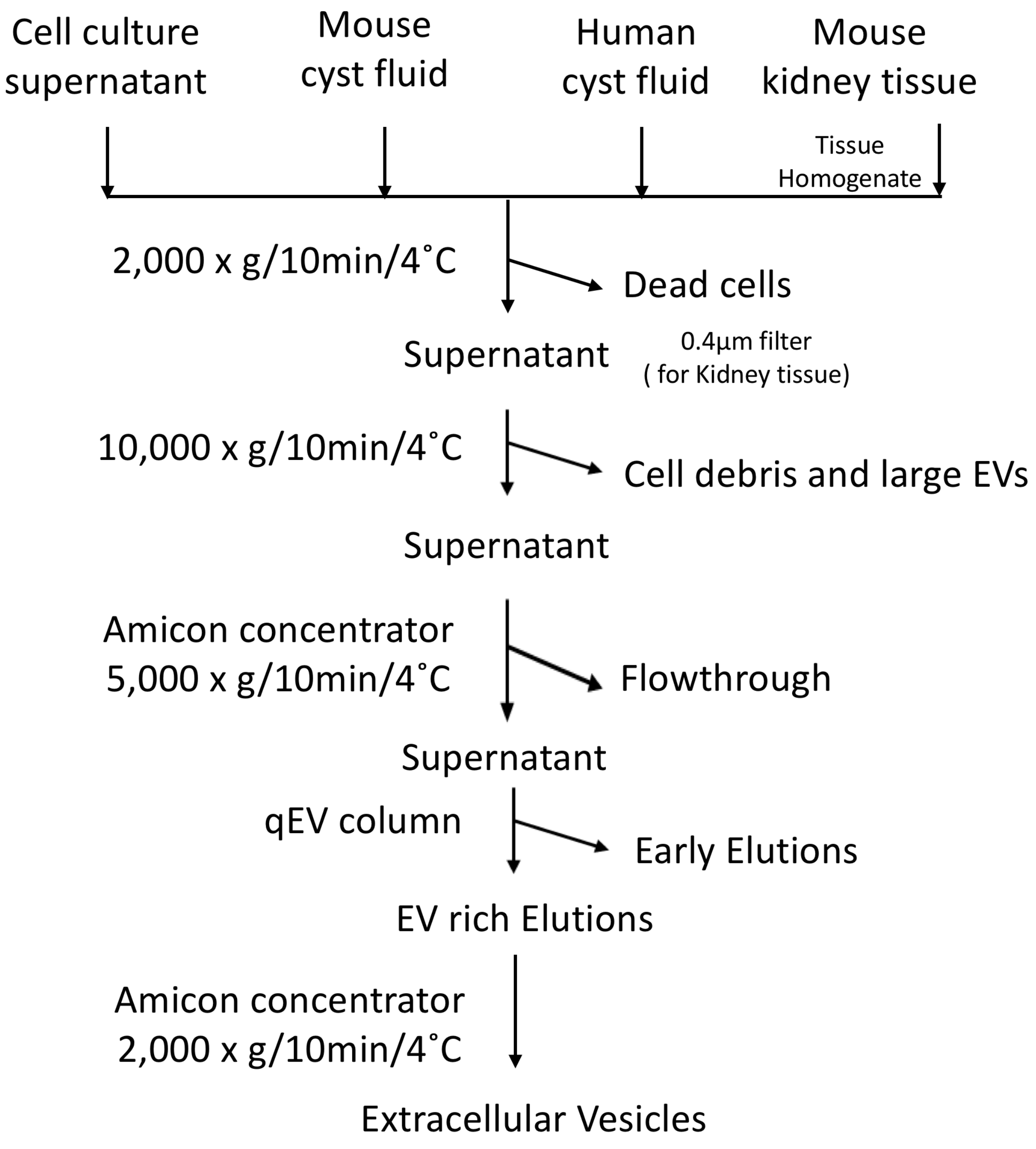

Supplementary Figure S2a: In vitro trafficking - *Tsc*

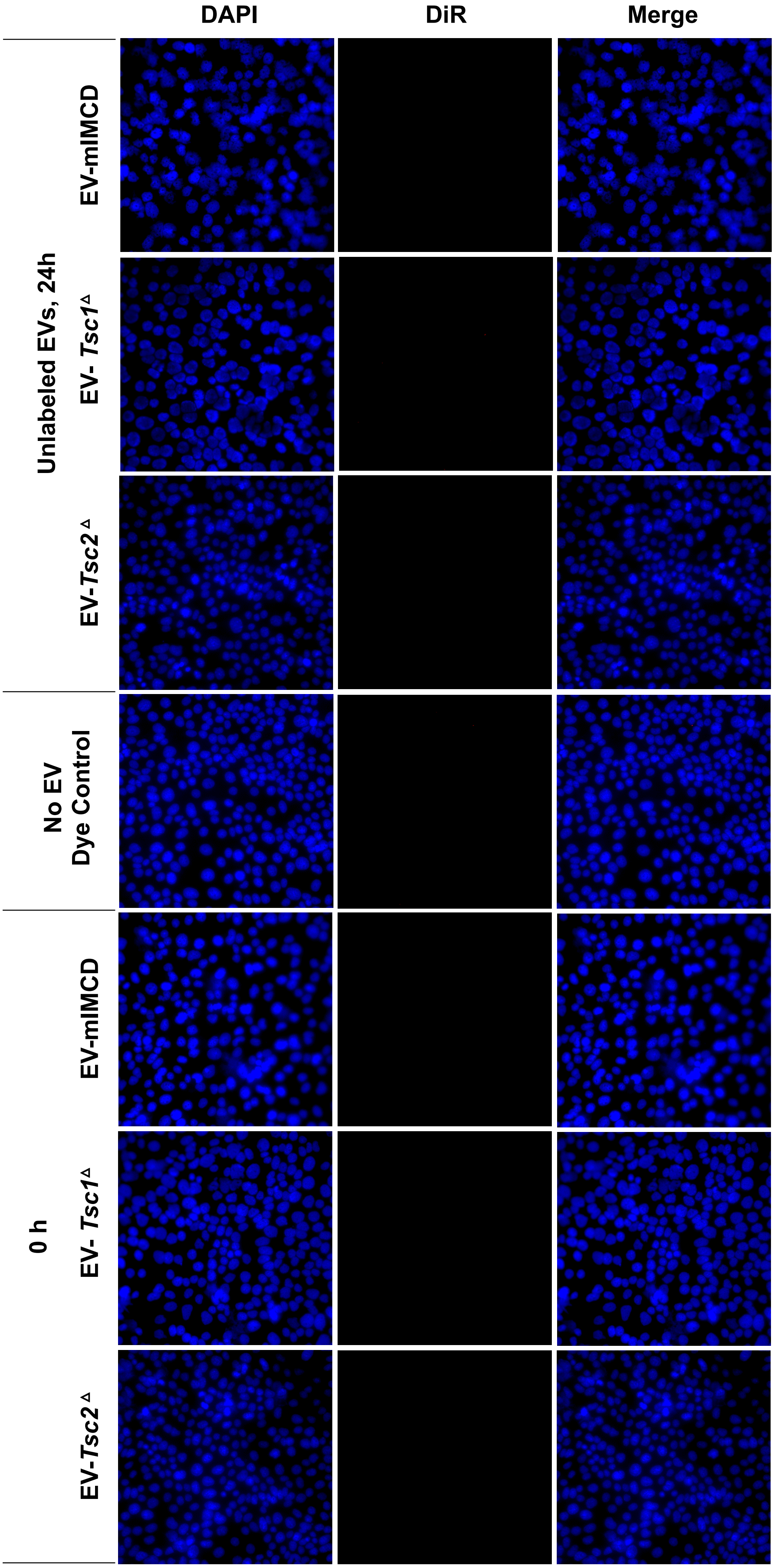

Supplementary Figure S2b: In vitro trafficking - *Tsc*

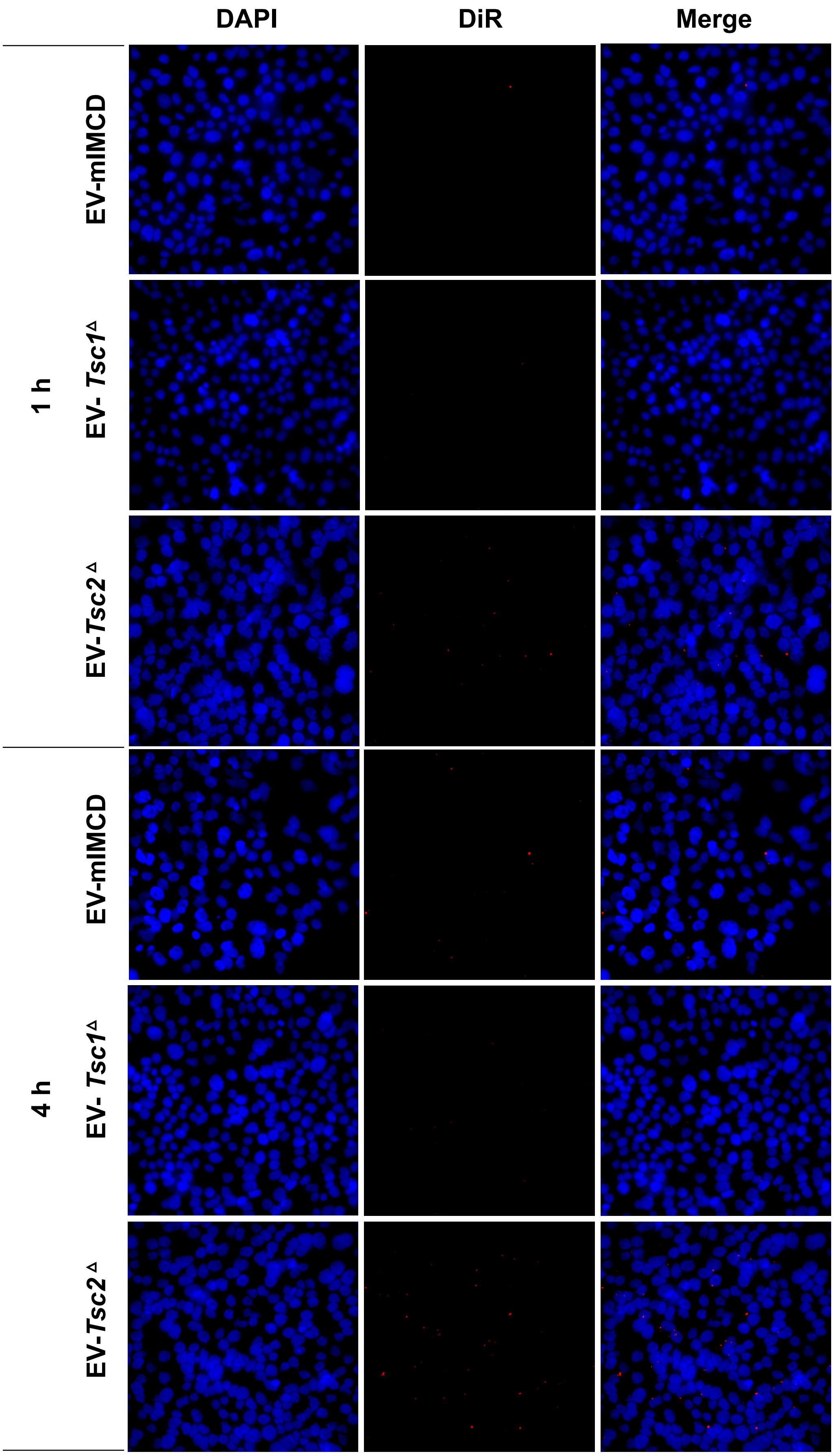

Supplementary Figure S2c: In vitro trafficking - *Tsc*

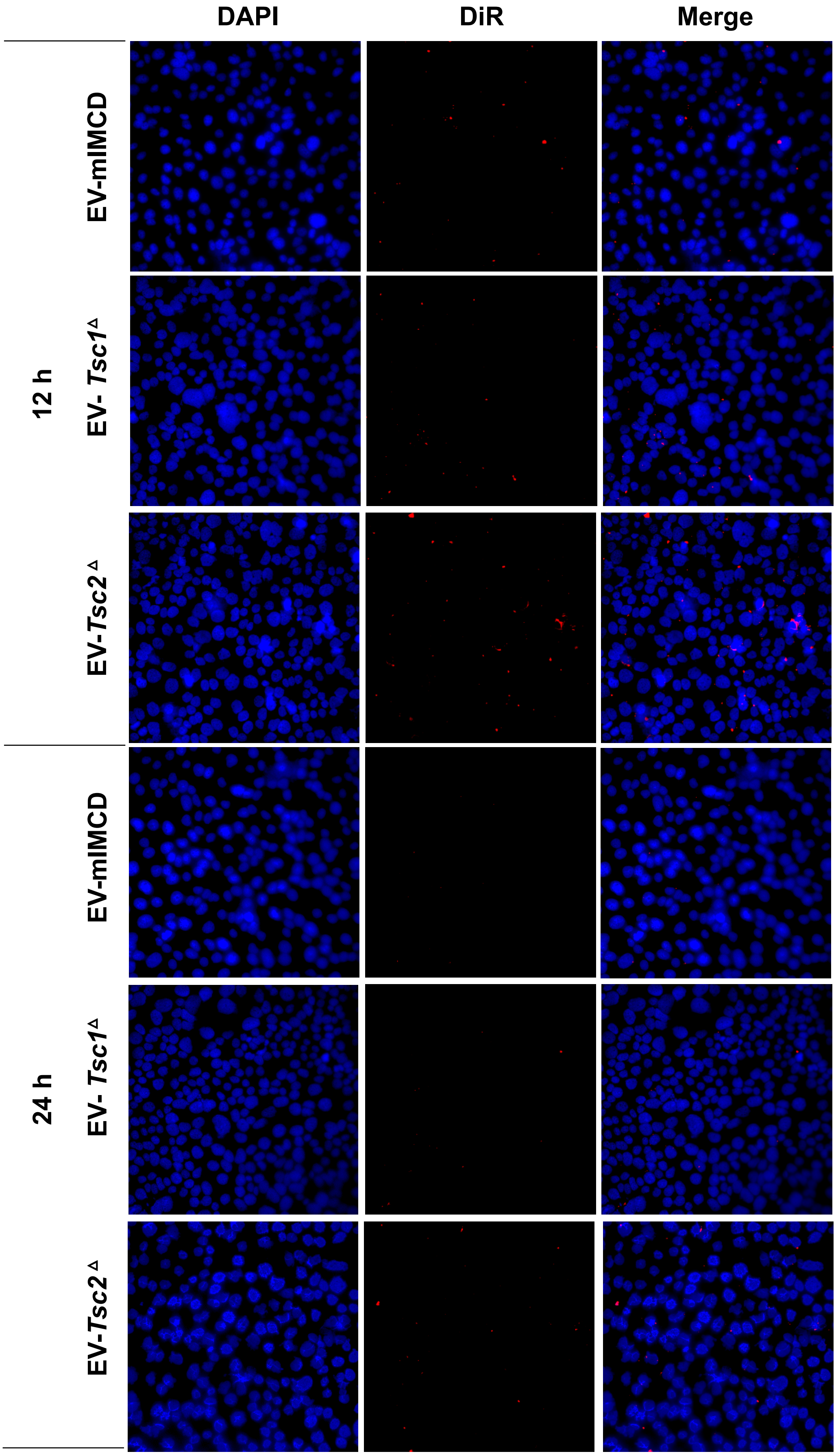

Supplementary Figure S2d: In vitro trafficking - *pkd*

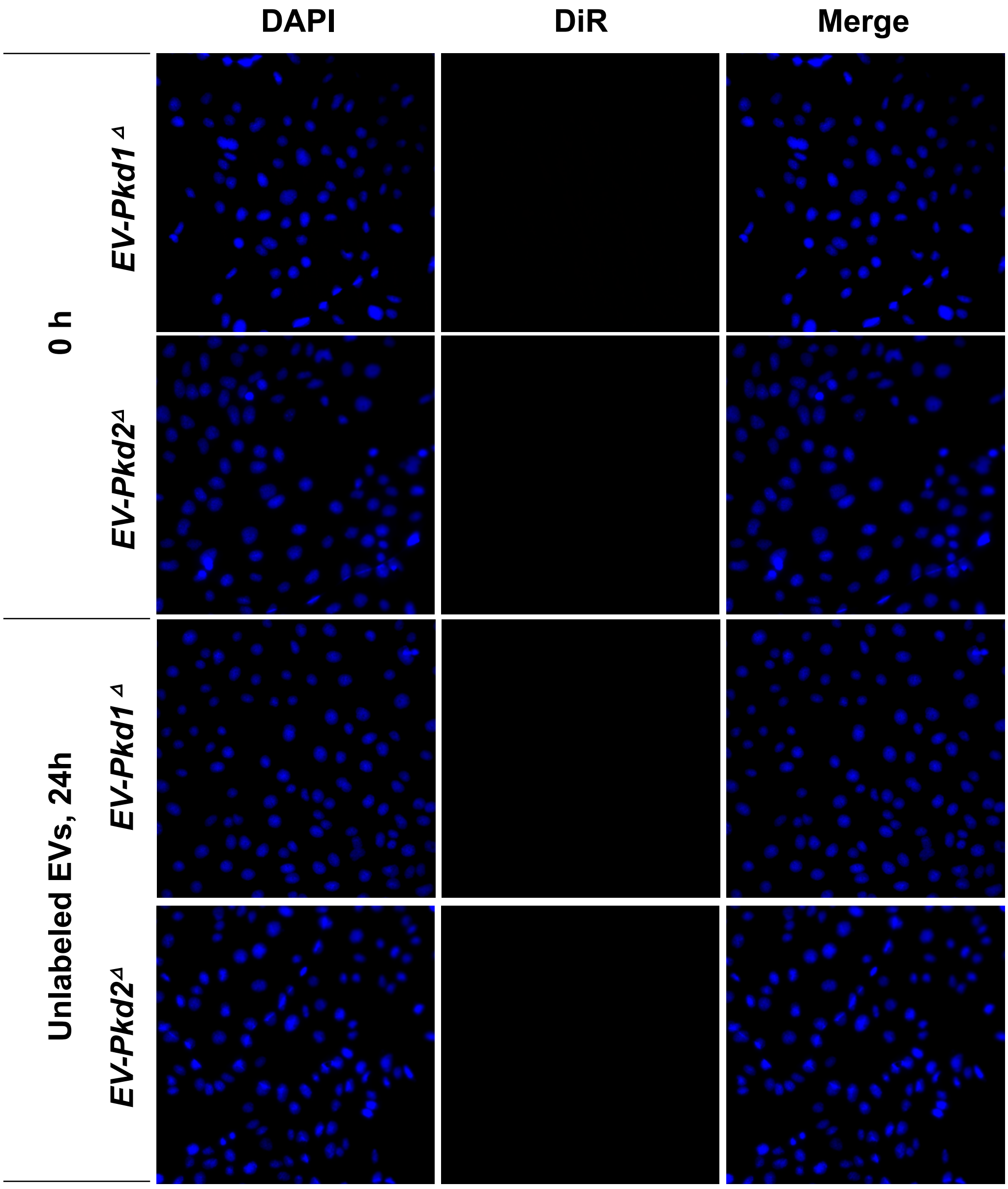

Supplementary Figure S2e: In vitro trafficking - *pkd*

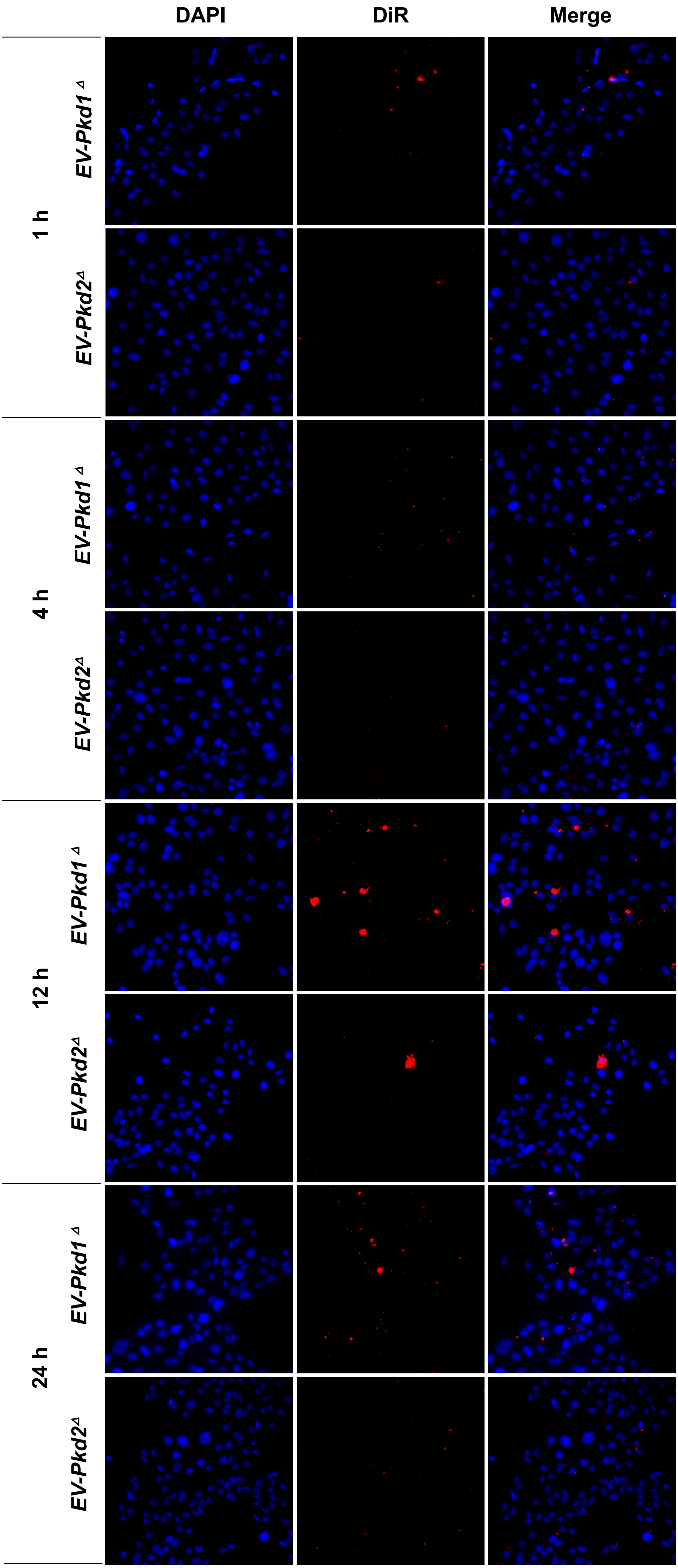

Supplement: Supplementary file 1 [file biology-11-00709-s001.zip › biology-1622955 supplementary Figure S1 and S2.pdf]
