# Supplementary figures and images for: Single Gene Mutations in Pkd1 or Tsc2 Alter Extracellular Vesicle Production and Trafficking
Source: Biology (Basel). 2022 May 6;11(5):709. doi: 10.3390/biology11050709 (PMC9139108; doi:10.3390/biology11050709)

## Slide 1
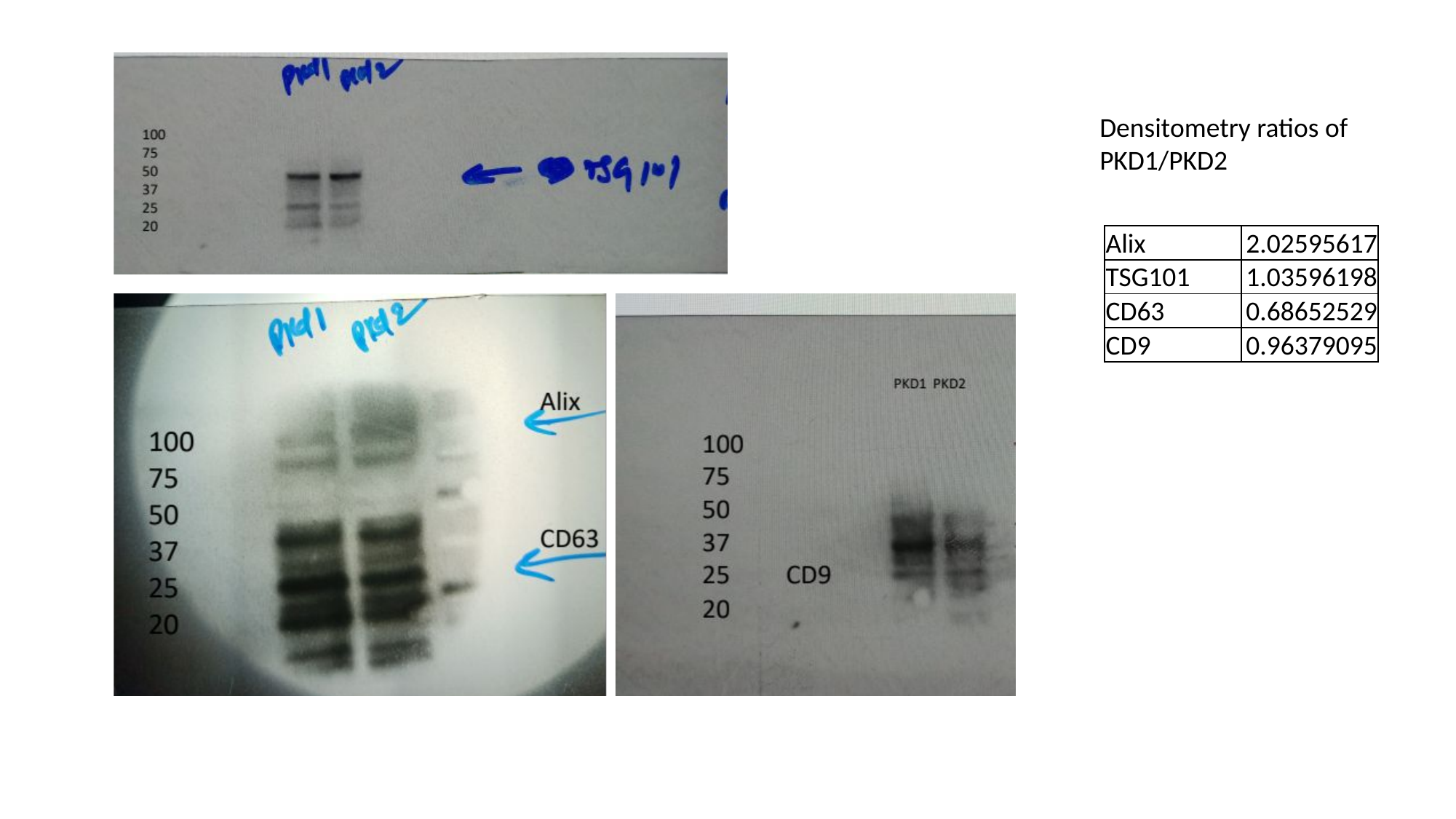

Densitometry ratios of PKD1/PKD2
| Alix | 2.02595617 |
| --- | --- |
| TSG101 | 1.03596198 |
| CD63 | 0.68652529 |
| CD9 | 0.96379095 |

Supplement: Supplementary file 1 [file biology-11-00709-s001.zip › Biology-1622955 File S1.pptx]
